# Previous treatment decreases efficacy of pralsetinib in RET fusion-positive non-small-cell lung cancer

**DOI:** 10.3389/fmed.2025.1467871

**Published:** 2025-01-24

**Authors:** Lei Wang, Yafei You, Wenzhuo He, Yu Hou, Lan Li, Li Wang, Chang Jiang, Jiahong Yi, Yaoxiong Xia, Liangping Xia

**Affiliations:** ^1^Department of Radiotherapy, The Third Affiliated Hospital of Kunming Medical University, Yunnan Cancer Hospital, Pecking University Cancer Hospital Yunnan, Kunming, Yunnan, China; ^2^The Department of Clinical Oncology, The Seventh Affiliated Hospital, Sun Yat-Sen University, Shenzhen, Guangdong, China; ^3^Department of VIP Region, Sun Yat-sen University Cancer Center, State Key Laboratory of Oncology in South China, Collaborative Innovation Center for Cancer Medicine, Guangzhou, Guangdong, China

**Keywords:** RET fusion, non-small-cell lung cancer, pralsetinib, survival outcomes, prognostic factors

## Abstract

**Background:**

Pralsetinib is a selective RET inhibitor. The ARROW trial revealed that RET fusion-positive non-small-cell lung cancer (NSCLC) can benefit from pralsetinib with tolerable adverse events (AEs). However, the efficacy and safety of pralsetinib in real world has rarely been reported.

**Materials and methods:**

This study reviewed the efficacy and safety of pralsetinib in RET fusion-positive NSCLC patients between March 2021 and December 2021. Progression-free survival (PFS) and overall survival (OS) were evaluated by a Kaplan-Meier analysis and log-rank test. A Cox regression model was performed to identify independent prognostic factors.

**Results:**

A total of 28 patients were enrolled, and the median follow-up time was 18.1 months. The objective response rate and disease control rate of the whole cohort were 57.2% and 71.4%, respectively, and the median PFS and OS were 8.1 months [95% confidence interval (CI), 3.1–13.2] and 13.8 months (95% CI, 2.8–24.8), respectively. Baseline characteristics of the treatment naive group and pre-treated group were listed. The median PFS tended to be better in treatment naive group (18.3 vs. 8.0 months, *P* = 0.067), while the median OS were similar between the two groups (28.4 vs. 11.6 months, *P* = 0.308). Patients with Eastern Cooperative Oncology Group Performance Status (ECOG PS) score of 2 had worse median PFS comparing with those with ECOG PS score of 0–1 (3.8 vs. 12.6 months, *P* = 0.004). Besides, patients previously treated with platinum-based chemotherapy (PBC) also revealed worse median PFS comparing with those without previous PBC (8.0 vs. 18.6 months, *P* = 0.023). Furthermore, patients previously treated with anti-programmed death-1 (PD-1) antibody or multikinase inhibitors (MKIs) showed worse median OS compared with those without previous anti-PD-1 antibody (5.0 vs. 22.0 months, *P* = 0.002) or MKIs (6.2 vs. 28.4 months, *P* = 0.015). The most common AEs was increased aspartate aminotransferase (39.3%).

**Conclusion:**

Pralsetinib was effective in RET fusion-positive NSCLC with tolerable AEs in real-world practice. Efficacy of pralsetinib was decreased in patients previously treated with PBC, immunotherapy, or MKIs.

## Introduction

Non-small-cell lung cancer (NSCLC) is one of the most common causes of cancer-related deaths globally (1). The treatment approaches of NSCLC include surgery, radiotherapy, chemotherapy, immunotherapy, and target therapy alone or in combination, but the effectiveness of current therapies are not ideal. Therefore, development of new treatment is necessary.

About 1%−3% NSCLC patients have rearranged during transfection (RET) fusions, resulting in RET pathway activation (2). Multikinase inhibitors (MKIs) with anti-RET activities have been used in RET fusion-positive NSCLC in clinical practice, but the effectiveness is limited with obvious off-target toxicities (3–5). Selpercatinib (LOXO-292) and pralsetinib (BLU667) are highly selective inhibitors targeted to RET alterations. LIBRETTO-001 trial reported that the median progression-free survival (PFS) in RET fusion-positive NSCLC patients treated with selpercatinib was 17 months, and the overall response rate (ORR) in untreated and pretreated patients were 64% [95% confidence interval (CI), 54%−73%] and 85% (95% CI, 70%−94%), respectively (6). Further, the ARROW trial revealed median PFS of 17.1 months in the whole cohort of RET-altered NSCLC treated with pralsetinib (7). Besides, the ARROW trial also demonstrated a response rate of 73% (95% CI, 52%−88%) in treatment-naive subgroup, and 61% (95% CI, 50%−72%) in treated subgroup (7). Thus, selpercatinib and pralsetinib are approved for the treatment of NSCLC patients with RET fusions, and pralsetinib has been approved in China in 2021 (8).

Although real-world experience of pralsetinib in RET fusion-positive NSCLC has been reported in Italy (9), the experience of pralsetinib among Chinese population has been rarely reported. Therefore, the retrospective study was conducted to provide insight for clinical practice.

## Materials and methods

### Patient selection

In this retrospective study, patients with locally advanced or metastatic NSCLC between March 2021 and December 2021 at Sun Yat-sen University Cancer Center were reviewed. Inclusion criteria were as follows: (1) histologically confirmed as NSCLC; (2) locally advanced or metastatic disease (stage IIIB, IIIC, or IV unresectable disease); and (3) received at least one dose of pralsetinib. The exclusion criteria were as follows: (1) combination therapy of pralsetinib with other anti-tumor drugs; (2) lack of treatment data; and (3) lost follow-up. All clinical records and image information were reviewed. The study was approved by the Medical Ethics Committee of Sun Yat-sen University Cancer Center (B2024-130-01). Key data of this study has been uploaded onto the Research Data Deposit public platform.

### Statistical analysis

Tumor response was defined by the Response Evaluation Criteria in Solid Tumors version 1.1 (10). The ORR referred to the rate of complete response (CR) and partial response (PR), and the disease control rate (DCR) referred to the rate of CR, PR, and stable disease (SD). PFS was defined as the beginning of pralsetinib to disease progression or death, and overall survival (OS) was defined as the beginning of pralsetinib to death due to any cause. Adverse events (AEs) during treatment were assessed based on the Common Terminology Criteria for Adverse Events version 5.0 (11).

Continuous and categorical variables were compared by chi squared and Mann-Whitney U tests, respectively. Survival outcomes were evaluated by the Kaplan-Meier method and log-rank test. All tests were two sided and *P* < 0.05 was considered statistically significant. Statistical analyses were performed using SPSS 25.0 software.

## Results

### Patient characteristics

A total of 28 RET fusion-positive advanced NSCLC patients treated with pralsetinib monotherapy were identified (Table 1). The median age was 54 years (range, 28–80 years), and 13 (46.4%) patients were male. Eight (28.6%) patients were current or former smokers, and seven (25.0%) patients had an Eastern Cooperative Oncology Group Performance Status (ECOG PS) score of 2. Except for one patient of mucoepidermoidcarcinoma, another 27 patients were diagnosed with adenocarcinoma, and 8 (28.6%) patients had brain metastasis. The most common RET fusion partner was KIF5B (53.6%). More than half (19/28, 67.9%) patients were previously treated with platinum-based chemotherapy (PBC), five patients were previously treated with anti-programmed death-1 (PD-1) antibody, and nearly half (12/28, 41.9%) patients previously received multikinase inhibitors (MKIs). There were eight patients had pralsetinib as the first-line therapy, and 20 patients were pre-treated.

**Table 1:** Baseline characteristics.

**Characteristics**	**Number (*n* = 28, %)**	**Treatment naive (*n* = 8, %)**	**Pre-treated (*n* = 20, %)**	***P*-value**
**Age (years)**				
< 60	21 (75.0%)	6 (75.0%)	15 (75.0%)	1.000
≥60	7 (25.0%)	2 (25.0%)	5 (25.0%)	
**Gender**				
Female	15 (53.6%)	3 (37.5%)	12 (60.0%)	0.381
Male	13 (46.4%)	5 (62.5%)	8 (40.0%)	
**Smoking history**				
Current or former	8 (28.6%)	3 (37.5%)	5 (25.0%)	0.636
Never or unknown	20 (71.4%)	5 (62.5%)	15 (75.0%)	
**Histology**				
Adenocarcinoma	27 (96.4%)	8 (100.0%)	19 (95.0%)	0.862
Other	1 (3.6%)	0 (0.0%)	1 (5.0%)	
**ECOG PS**				
0–1	21 (75.0%)	7 (87.5%)	14 (70.0%)	0.500
2	7 (25.0%)	1 (12.5%)	6 (30.0%)	
**Brain metastasis**				
Yes	8 (28.6%)	1 (12.5%)	7 (35.0%)	0.381
No	20 (71.4%)	7 (87.5%)	13 (65.0%)	
**RET fusion partner**				
KIF5B	15 (53.6%)	4 (50.0%)	11 (55.0%)	0.784
CCDC6	5 (17.9%)	2 (25.0%)	3 (15.0%)	
Other	5 (17.9%)	0 (0.0%)	5 (25.0%)	
Unknown	3 (10.7%)	2 (25.0%)	1 (5.0%)	
**Lines of previous therapy**				
0	8 (28.6%)	8 (100.0%)	0 (0.0%)	/
1–2	14 (50.0%)	0 (0.0%)	14 (70.0%)	
≥3	6 (21.4%)	0 (0.0%)	6 (30.0%)	
**Previous therapy**				
PBC	19 (67.9%)	0 (0.0%)	19 (95.0%)	/
Anti-PD-1 antibody	5 (17.9%)	0 (0.0%)	5 (25.0%)	
MKIs	12 (42.9%)	0 (0.0%)	12 (60.0%)	

ECOG PS, Eastern Cooperative Oncology Group Performance Status; PBC, Platinum-based chemotherapy; PD-1, Programmed death-1; MKIs, Multikinase inhibitors.

### Treatment

All patients were initially treated with 400 mg once daily. Eight (28.6%) patients experienced dose reduction due to AEs, including one patient of hepatotoxicity, two patients of decreased platelets, one patient of increased creatinine, two patients of increased alkaline phosphatase and aspartate aminotransferase, one patient of pneumonitis, and one patient of musculoskeletal pain, respectively. Besides, three (10.7%) patients discontinued the therapy because of hepatotoxicity, pulmonary fibrosis, and financial reason, respectively. Therapies after pralsetinib were as follows: rechallenge of chemotherapy with or without bevacizumab, local therapy, and immunotherapy.

### Efficacy

**Figure 1:**
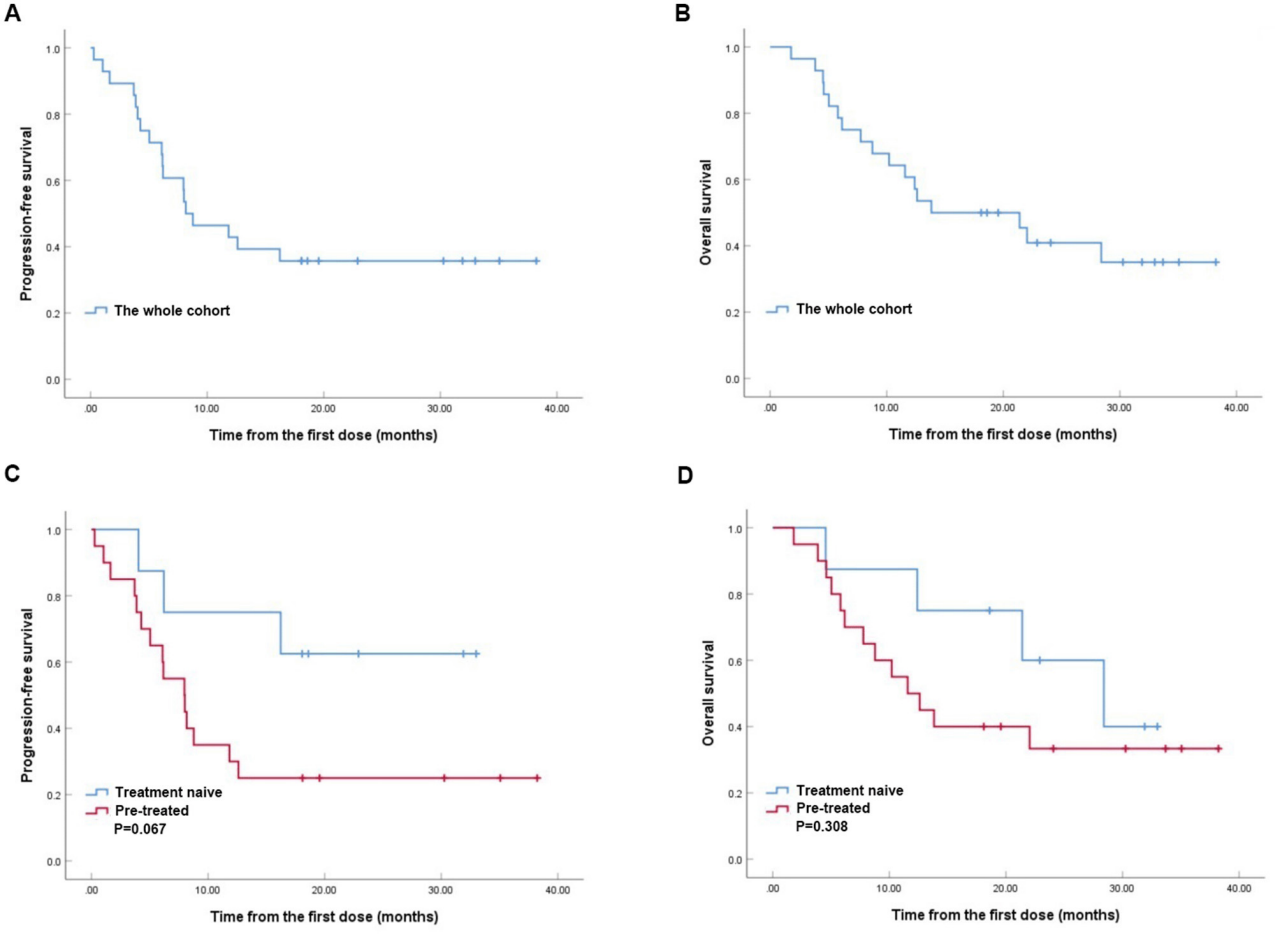
Kaplan–Meier analyses of progression-free survival (PFS) **(A)** and overall survival (OS) **(B)** in RET fusion-positive non-small-cell lung cancer patients treated with pralsetinib. Kaplan-Meier analyses of PFS **(C)** and OS **(D)** between treatment naive group and pre-treated group.

With a median follow-up time of 18.1 months (range, 1.8–38.2) of the whole cohort, the median PFS and OS were 8.1 months (95% CI, 3.1–13.2; Figure 1A) and 13.8 months (95% CI, 2.8–24.8; Figure 1B), respectively. The median PFS tended to be better in treatment naive group compared with the pre-treated group (18.3 vs. 8.0 months, *P* = 0.067, Figure 1C), while the median OS were similar between the two groups (28.4 vs. 11.6 months, *P* = 0.308, Figure 1D).

In the whole cohort, The ORR and DCR rates were 57.2 and 71.4%, respectively (Table 2). One (3.6%) patient achieved CR, 15 (53.6%) patients achieved PR, four (14.3%) patients revealed SD, six (21.5%) patients developed progressive disease, and 2 (7.1%) patients could not be evaluated. Although the best overall responses were similar between the treatment naive group and the pre-treated group (*P* = 0.443), the treatment naive group gained better median duration of response (*P* = 0.050).

**Table 2:** Clinical activity endpoints in patients with measurable disease.

**Variables**	**Number (%)**	**Treatment naive (*n* = 8, %)**	**Pre-treated (*n* = 20, %)**	***P*-value**
Overall response rate	16 (57.2%)	6 (75.0%)	14 (70.0%)	/
Disease control rate	20 (71.4%)	6 (75.0%)	19 (95.0%)	/
**Best overall response**				
Complete response	1 (3.6%)	0 (0.0%)	1 (5.0%)	0.443
Partial response	15 (53.6%)	6 (75.0%)	8 (40.0%)	
Stable disease	4 (14.3%)	0 (0.0%)	5 (25.0%)	
Progressive disease	6 (21.4%)	1 (12.5%)	5 (25.0%)	
Not evaluable	2 (7.1%)	1 (12.5%)	1 (5.0%)	
**Median duration of response, months**				
Rate at 6 months	71.4%	87.5%	65.0%	0.050
Rate at 12 months	42.9%	75.0%	30.0%	

### Univariate and multivariate analysis

Univariate and multivariate analysis were conducted to identify independent prognostic factors (Table 3). The univariate and multivariate analysis showed that poor ECOG PS [hazard ratio (HR), 5.052; 95% CI, 1.595–16.008; *P* = 0.006] and previous PBC (HR, 4.320; 95% CI, 1.111–16.797; *P* = 0.035) were independent prognostic factors in PFS, while previous anti-PD-1 antibody (HR, 3.168; 95% CI, 1.010–9.941; *P* = 0.048) and previous MKIs (HR, 3.777; 95% CI, 1.284–11.111; *P* = 0.016) were independent prognostic factors in OS. Furthermore, no statistical significance of brain metastasis in PFS (HR, 0.917; 95% CI, 0.326–2.576; *P* = 0.869) and OS (HR, 0.693; 95% CI, 0.225–2.131; *P* = 0.522) were observed.

**Table 3:** Univariate and multivariate analysis of prognostic factors for 28 patients treated with pralsetinib.

**Variables**	**PFS**				**OS**			
	**Univariate analysis**		**Multivariate analysis**		**Univariate analysis**		**Multivariate analysis**	
	**HR (95% CI)**	***P***	**HR (95% CI)**	***P***	**HR (95% CI)**	***P***	**HR (95% CI)**	***P***
**Age (years)**								
< 60	Ref.		–		Ref.		–	
≥60	2.076 (0.758–5.682)	0.155			1.963 (0.719–5.361)	0.188		
**Gender**								
Female	Ref.		–		Ref.		–	
Male	1.002 (0.394–2.549)	0.996			0.745 (0.287–1.938)	0.547		
**Smoking history**								
Current or former	Ref.		–		Ref.		–	
Never or unknown	1.031 (0.367–2.898)	0.954			1.490 (0.548–4.049)	0.435		
**ECOG PS**								
0–1	Ref.		**Ref**.		Ref.		–	
2	3.983 (1.450–10.940)	0.007	**5.052 (1.595–16.008)**	**0.006**	2.616 (0.895–7.645)	0.079		
**Brain metastasis**								
Yes	Ref.		–		Ref.		–	
No	0.917 (0.326–2.576)	0.869			0.693 (0.225–2.131)	0.522		
**Lines of previous therapy**								
0	Ref.				Ref.		–	
1–2	2.982 (0.823–10.801)	0.096			1.536 (0.472–5.000)	0.476		
≥3	3.177 (0.707–14.272)	0.131			2.955 (0.705–12.386)	0.138		
**Previous PBC**								
Yes	Ref.		**Ref**.		Ref.		–	
No	3.884 (1.110–13.588)	0.034	**4.320 (1.111–16.797)**	**0.035**	20320 (0.748–7.193)	0.145		
**Previous anti-PD-1 antibody**								
Yes	Ref.				Ref.		**Ref**.	
No	3.189 (1.065–9.548)	0.038	1.782 (0.554–5.735)	0.333	5.021 (1.634–15.426)	0.005	**3.168 (1.010–9.941)**	**0.048**
**Previous MKIs**								
Yes	Ref.				Ref.		**Ref**.	
No	1.924 (0.760–4.871)	0.167			3.239 (1.201–8.736)	0.020	**3.777 (1.284–11.111)**	**0.016**

HR, hazard ratio; CI, confidence interval; ECOG PS, Eastern Cooperative Oncology Group Performance Status; PBC, Platinum-based chemotherapy; PD-1, Programmed death-1; MKIs, Multikinase inhibitors. The bold values were used to highlight the statistical significance.

### Subgroup analysis

**Figure 2:**
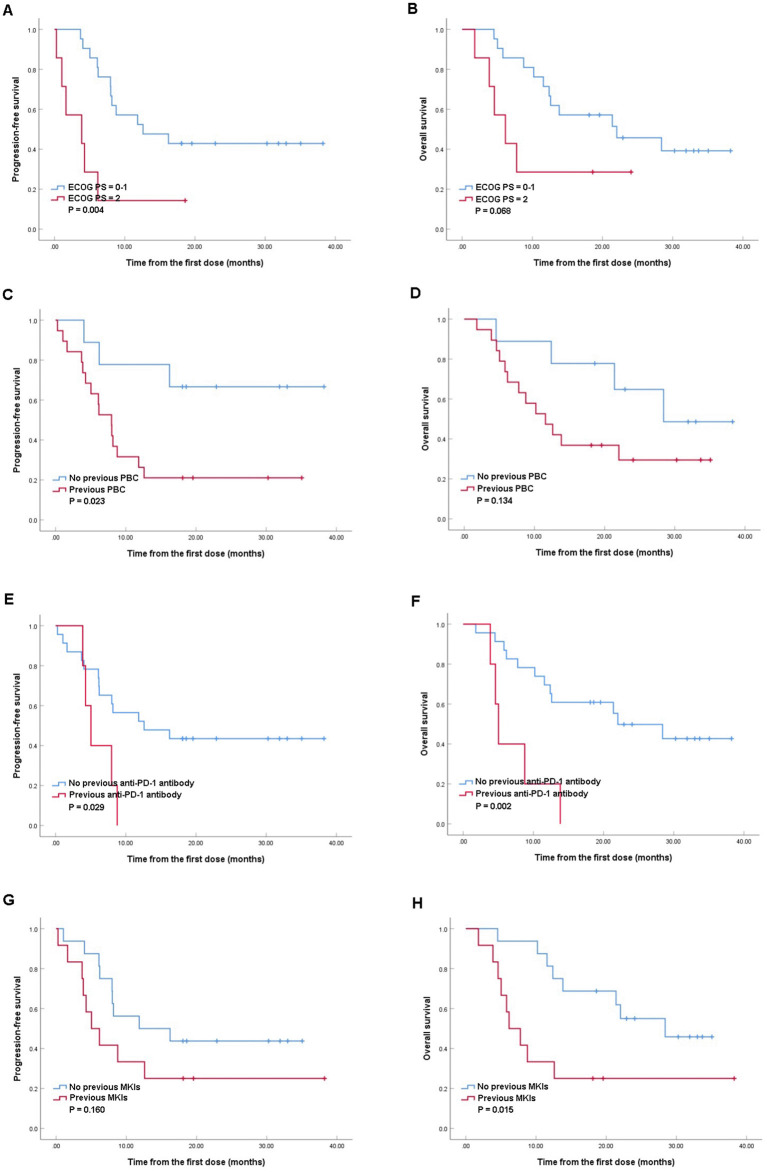
Kaplan–Meier analyses of progression-free survival and overall survival in patients with Eastern Cooperative Oncology Group Performance Status (ECOG PS) of 0–1 and ECOG PS of 2 **(A, B)**; patients with or without previous platinum-based chemotherapy (PBC) **(C, D)**, patients with or without previous anti-programmed death-1 (PD-1) antibody **(E, F)**, patients with or without previous multikinase inhibitors (MKIs) **(G, H)**.

Further analysis of PFS and OS in different subgroups were conducted (Figure 2), and the baseline characteristics between the compared groups were listed in Supplementary material (Tables 1–4). Significant better median PFS was observed in patients with ECOG PS score of 0–1 comparing with those with ECOG PS score of 2 (12.6 vs. 3.8 months, *P* = 0.004; Figure 2A), while the median OS tended to be better in ECOG PS score of 0–1 group (22.0 vs. 6.2 months, *P* = 0.068; Figure 2B). Besides, patients without previous PBC gained longer median PFS comparing with those previously treated with PBC (18.6 vs. 8.0 months, *P* = 0.023; Figure 2C), while the median OS was similar between the two groups (28.4 vs. 11.6 months, *P* = 0.134; Figure 2D). Moreover, better median PFS (12.6 vs. 5.0 months, *P* = 0.029; Figure 2E) and OS (22.0 vs. 5.0 months, *P* = 0.002; Figure 2F) were observed in patients without previous use of anti-PD-1 antibody comparing with those with previous anti-PD-1 antibody. Furthermore, although similar median PFS were observed in patients with or without previous MKIs (5.0 vs. 11.8 months, *P* = 0.160; Figure 2G), patients without previous use of MKIs revealed better median OS (28.4 vs. 6.2 months, *P* = 0.015; Figure 2H).

### Safety

AEs during treatment are listed in Table 4. The most common hematological AEs were decreased hemoglobin (32.1%), decreased neutrophils (32.1%), and decreased platelets (14.3%). Increased aspartate aminotransferase (AST) and increased alanine aminotransferase (ALT) occurred in 39.3% and 25.0% patients, respectively, with 1 patient suffering from Grade 3 increased ALT. Decreased sodium affected 10.7% patients, with one patient experiencing ≥ Grade 3 decreased sodium. The most common general AEs were hypertension (3/28, 10.7%), vomiting (3/28, 10.7%), and pneumonitis (3/28, 10.7%), while one patient had ≥ Grade 3 hypertension and 1 patient had ≥ Grade 3 vomiting. Moreover, no patients had diarrhea or constipation during the treatment.

**Table 4:** Treatment-related adverse events.

**Adverse events**	**Any grade (%)**	**Grade 3–4 (%)**
**Hematology**		
Decreased hemoglobin	9 (32.1%)	1 (3.6%)
Decreased lymphocytes	1 (3.6%)	0 (0.0%)
Decreased neutrophils	9 (32.1%)	1 (3.6%)
Decreased platelets	4 (14.3%)	1 (3.6%)
**Chemistry**		
Increased AST	11 (39.3%)	0 (0.0%)
Increased ALT	7 (25.0%)	1 (3.6%)
Increased creatinine	1 (3.6%)	0 (0.0%)
Increased alkaline phosphatase	2 (7.1%)	0 (0.0%)
Decreased calcium (corrected)	0 (0.0%)	0 (0.0%)
Decreased sodium	3 (10.7%)	1 (3.6%)
**General**		
Fatigue	2 (7.1%)	0 (0.0%)
Pyrexia	1 (3.6%)	0 (0.0%)
Edema	3 (10.7%)	0 (0.0%)
Musculoskeletal pain	1 (3.6%)	0 (0.0%)
Hypertension	3 (10.7%)	1 (3.6%)
Dry mouth	1 (3.6%)	0 (0.0%)
Diarrhea	0 (0.0%)	0 (0.0%)
Constipation	0 (0.0%)	0 (0.0%)
Vomiting	3 (10.7%)	1 (3.6%)
Pneumonitis	3 (10.7%)	0 (0.0%)

AST, aspartate aminotransferase; ALT, alanine aminotransferase.

## Discussion

RET fusions were firstly identified in lung cancer in 2012 (12), and MKIs such as cabozantinib (3), lenvatinib (4), and vandetanib (5) were available with limited responses and high rates of off-target toxicities. Pralsetinib is a highly selective RET inhibitor being developed for the treatment of various solid tumors with RET fusions (8). The phase 1/2 ARROW study enrolled 233 patients with locally advanced or metastatic solid RET fusion-positive NSCLC to treat with 400 mg once-daily oral pralsetinib (7). In the previously-treated cohort, the ORR was 57%, including 5/87 (5.7%) CR and 48/87 (55%) PR, respectively, while the ORR was 70% in the treatment-naive cohort, including 3/27 (11%) CR and 16/27 (59%) PR, respectively (7). Therefore, pralsetinib was approved for the treatment of RET fusion-positive NSCLC in the United States in 2020, and in China in 2021 (8).

In our real-world study, 28 patients with RET fusion-positive NSCLC were treated with pralsetinib monotherapy. Consistent to previous investigations demonstrating that RET fusion revealed higher frequencies in younger non-smoking female with lung adenocarcinoma (2, 13, 14), the present study showed that 75% patients had age of < 60 years old, more than half patients were female, and 71.4% patients were never or unknown smokers. Besides, we observed that the most common RET fusions were KIF5B-RET (53.6%) and CCDC6-RET (17.9%), which was similar to the clinical trials (6, 7). However, the proportion of patients with ECOG PS score of 2 was 25% in the current study, which was higher than prospective studies (6, 7). Furthermore, similar to real-world data from Italy (9), only around 20% patients received pralsetinib as their first-line treatment in the current study, which was different from the analysis of the part of phase 1/2 ARROW trial in China including 31 patients in the treatment naive group and 37 patients in previous platinum-based chemotherapy group (15). Although no significant difference was observed in median OS between treatment naive group and pre-treated group (28.4 vs. 11.6 months, *P* = 0.308), treatment naive group tended to reveal longer median PFS (18.3 vs. 8.0 months, *P* = 0.067). Thus, we proposed clinicians to conduct gene test in advanced NSCLC at diagnosis, and to use pralsetinib as the first-line therapy in patients with RET fusion.

Moreover, in this retrospective study, similar ORR among the current study (57.2%), the ARROW trial (53.0%) (7), the Chinese population of the ARROW trial (66.7%) (16), and real-world investigation from Italy (66.0%) (9) were observed. However, we observed similar median PFS in the present study comparing with the real-world investigation in Italy (8.1 vs. 8.9 months) (9), but shorter median PFS comparing with the ARROW trial (8.1 vs. 17.1 months) (7) and the Chinese population analysis in ARROW trial (8.1 VS. 11.7 months) (15). These results demonstrated that the efficacy of pralsetinib in real world might be influenced by other risk factors such as previous treatments, and further investigations are warrant.

Furthermore, we observed statistical significance of previous anti-PD-1 antibody in PFS in univariate analysis (HR, 3.189; 95% CI, 1.065–9.0548; *P* = 0.038), and identified previous anti-PD-1 antibody as independent prognostic factor in OS (HR, 3.168; 95% CI, 1.010–9.941; *P* = 0.048). Besides, previous use of MKIs was identified as an independent prognostic factor in OS (HR, 3.777; 95% CI, 1.284–11.111; *P* = 0.016). These results were consistent to which reported by Meng et al. (16), indicating that patients with RET fusion NSCLC are not likely to benefit well from immunotherapy and MKIs. We also observed no statistical significance of brain metastasis in PFS (HR, 0.917; 95% CI, 0.326–2.576; *P* = 0.869) and OS (HR, 0.693; 95% CI, 0.225–2.131; *P* = 0.522) in the present study. Subbiah et al. investigated the intracranial efficacy of selpercatinib, and observed an ORR of 82% and an CR of 23% among 22 patients with measurable intracranial disease, showing a robust and durable intracranial efficacy in RET fusion-positive NSCLC patients (17). Pralsetinib showed blood penetration and activity against intracranial tumors in preclinical model (18), and Passaro et al. (9) reported effectiveness of pralsetinib in intracranial disease with ORR of 66.7%. Therefore, we preferred to propose the efficacy of pralsetinib in patients with brain metastasis, and more head-to-head comparisons and large sample real-world studies are needed.

There are some differences between our study and the ARROW trial in terms of the AEs profile. Neutropenia (21%) was the most common hematological side effect in the ARROW trial (7), while decreased hemoglobin (32.1%) and neutrophils (32.1%) were the most common AEs in the current study. We preferred to attribute this difference to poor performance status (ECOG PS = 2, 25.0%) and heavy previous treatment (lines ≥3, 21.4%) before the use of pralsetinib in our study. In addition, the incidence of increased creatinine in our investigation (3.6%) was lower than which in the ARROW trial (8%) (7), while the incidences of increased AST and ALT were similar between our study and the ARROW trial. This might result from that pralsetinib is mainly metabolized by liver (8).

The present study had some limitations. This was a retrospective study based on experience from a single institution with a small sample size. Although bias was unavoidable, we collected detailed data to reveal the treatment of pralsetinib in advanced NSCLC with RET fusions in real world, in order to provide clinical reference for future treatment.

## Conclusions

Our study demonstrated that pralsetinib was effective in RET fusion-positive advanced NSCLC with tolerable AEs. The benefits of pralsetinib in patients previously treated with PBC, immunotherapy, or MKIs were decreased. Since this is a retrospective study from a single institution with small sample size, further large sample real-world studies worldwide are warranted.

## Data Availability

The raw data supporting the conclusions of this article are available upon request to the authors, or from the Research Data Deposit public platform (https://www.researchdata.org.cn).
