# Supplementary material for: Previous treatment decreases efficacy of pralsetinib in RET fusion-positive non-small-cell lung cancer
Source: Front Med (Lausanne). 2025 Jan 24;12:1467871. doi: 10.3389/fmed.2025.1467871 (PMC11804255; doi:10.3389/fmed.2025.1467871)
Supplement: Supplementary file 1 [file Table_1.doc]

**Table 1. Baseline characteristics between patients with ECOG PS=0-1 and ECOG PS =2**

| Characteristics | Number (n=28, %) | ECOG PS=0-1 (n=21, %) | ECOG PS=2 (n=7, %) | P value |
| --- | --- | --- | --- | --- |
| Age (years) |  |  |  | 0.604 |
| <60 | 21 (75.0%) | 15 (71.4%) | 6 (85.7%) |  |
| ≥60 | 7 (25.0%) | 6 (28.6%) | 1 (14.3%) |  |
| Gender |  |  |  | 0.376 |
| Female | 15 (53.6%) | 10 (47.6%) | 5 (71.4%) |  |
| Male | 13 (46.4%) | 11 (52.4%) | 2 (28.6%) |  |
| Smoking history |  |  |  | 0.466 |
| Current or former | 8 (28.6%) | 7 (33.3%) | 1 (14.3%) |  |
| Never or unknown | 20 (71.4%) | 14 (66.7%) | 6 (85.7%) |  |
| Histology |  |  |  | 0.604 |
| Adenocarcinoma | 27 (96.4%) | 21 (100.0%) | 6 (85.7%) |  |
| Other | 1 (3.6%) | 0 (0.0%) | 1 (14.3%) |  |
| Brain metastasis |  |  |  | 0.466 |
| Yes | 8 (28.6%) | 7 (33.3%) | 1 (14.3%) |  |
| No | 20 (71.4%) | 14 (66.7%) | 6 (85.7%) |  |
| RET fusion partner |  |  |  | 0.113 |
| KIF5B | 15 (53.6%) | 9 (42.9%) | 6 (85.7%) |  |
| CCDC6 | 5 (17.9%) | 5 (23.8%) | 0 (0.0%) |  |
| Other | 5 (17.9%) | 4 (19.0%) | 1 (14.3%) |  |
| Unknown | 3 (10.7%) | 3 (14.3%) | 0 (0.0%) |  |
| Lines of previous therapy |  |  |  | 0.055 |
| 0 | 8 (28.6%) | 7 (33.3%) | 1 (14.3%) |  |
| 1-2 | 14 (50.0%) | 12 (57.2%) | 2 (28.6%) |  |
| ≥3 | 6 (21.4%) | 2 (9.5%) | 4 (57.1%) |  |
| Previous therapy |  |  |  |  |
| PBC | 19 (67.9%) | 13 (61.9%) | 6 (85.7%) | 0.376 |
| Anti-PD-1 antibody | 6 (21.4%) | 3 (14.3%) | 2 (28.6%) | 0.717 |
| MKIs | 12 (42.9%) | 7 (33.3%) | 5 (71.4%) | 0.140 |

ECOG PS, Eastern Cooperative Oncology Group Performance Status; PBC, Platinum-based chemotherapy; PD-1, Programmed death-1; MKIs, Multikinase inhibitors.

**Table 2. Baseline characteristics between patients with or without previous PBC**

| Characteristics | Number (n=28, %) | Previous PBC (n=19, %) | No previous PBC (n=9, %) | P value |
| --- | --- | --- | --- | --- |
| Age (years) |  |  |  | 0.885 |
| <60 | 21 (75.0%) | 14 (73.7%) | 7 (77.8%) |  |
| ≥60 | 7 (25.0%) | 5 (26.3%) | 2 (22.2%) |  |
| Gender |  |  |  | 0.595 |
| Female | 15 (53.6%) | 11 (57.9%) | 4 (44.4%) |  |
| Male | 13 (46.4%) | 8 (42.1%) | 5 (55.6%) |  |
| Smoking history |  |  |  | 0.772 |
| Current or former | 8 (28.6%) | 5 (26.3%) | 3 (33.3%) |  |
| Never or unknown | 20 (71.4%) | 14 (73.7%) | 6 (66.7%) |  |
| Histology |  |  |  | 0.847 |
| Adenocarcinoma | 27 (96.4%) | 18 (94.7%) | 9 (100.0%) |  |
| Other | 1 (3.6%) | 1 (5.3%) | 0 (0.0%) |  |
| ECOG PS |  |  |  | 0.410 |
| 0-1 | 21 (75.0%) | 13 (68.4%) | 8 (88.9%) |  |
| 2 | 7 (25.0%) | 6 (31.6%) | 1 (11.1%) |  |
| Brain metastasis |  |  |  | 0.285 |
| Yes | 8 (28.6%) | 7 (36.8%) | 1 (11.1%) |  |
| No | 20 (71.4%) | 12 (63.2%) | 8 (88.9%) |  |
| RET fusion partner |  |  |  | 0.498 |
| KIF5B | 15 (53.6%) | 11 (57.9%) | 4 (44.4%) |  |
| CCDC6 | 5 (17.9%) | 3 (15.8%) | 2 (22.2%) |  |
| Other | 5 (17.9%) | 4 (21.1%) | 1 (11.1%) |  |
| Unknown | 3 (10.7%) | 1 (5.3%) | 2 (22.2%) |  |
| Previous therapy |  |  |  |  |
| Anti-PD-1 antibody | 6 (21.4%) | 5 (26.3%) | 0 (0.0%) | 0.530 |
| MKIs | 12 (42.9%) | 11 (57.9%) | 1 (1.11%) | 0.048 |

ECOG PS, Eastern Cooperative Oncology Group Performance Status; PBC, Platinum-based chemotherapy; PD-1, Programmed death-1; MKIs, Multikinase inhibitors.

**Table 3. Baseline characteristics between patients with or without previous anti-PD-1 antibody**

| Characteristics | Number (n=28, %) | Previous PD-1 inhibitor (n=5, %) | No previous PD-1 inhibitor (n=23, %) | P value |
| --- | --- | --- | --- | --- |
| Age (years) |  |  |  | 0.862 |
| <60 | 21 (75.0%) | 4 (80.0%) | 17 (73.9%) |  |
| ≥60 | 7 (25.0%) | 1 (20.0%) | 6 (26.1%) |  |
| Gender |  |  |  | 0.290 |
| Female | 15 (53.6%) | 4 (80.0%) | 11 (47.8%) |  |
| Male | 13 (46.4%) | 1 (20.0%) | 12 (52.2%) |  |
| Smoking history |  |  |  | 0.727 |
| Current or former | 8 (28.6%) | 1 (20.0%) | 7 (30.4%) |  |
| Never or unknown | 20 (71.4%) | 4 (80.0%) | 16 (69.6%) |  |
| Histology |  |  |  | 0.908 |
| Adenocarcinoma | 27 (96.4%) | 5 (100.0%) | 22 (95.7%) |  |
| Other | 1 (3.6%) | 0 (0.0%) | 1 (4.3%) |  |
| ECOG PS |  |  |  | 0.560 |
| 0-1 | 21 (75.0%) | 3 (60.0%) | 18 (78.3%) |  |
| 2 | 7 (25.0%) | 2 (40.0%) | 5 (21.7%) |  |
| Brain metastasis |  |  |  | 0.641 |
| Yes | 8 (28.6%) | 3 (60.0%) | 6 (26.1%) |  |
| No | 20 (71.4%) | 2 (40.0%) | 17 (73.9%) |  |
| RET fusion partner |  |  |  | 0.684 |
| KIF5B | 15 (53.6%) | 3 (60.0%) | 12 (52.2%) |  |
| CCDC6 | 5 (17.9%) | 1 (20.0%) | 4 (17.4%) |  |
| Other | 5 (17.9%) | 1 (20.0%) | 4 (17.4%) |  |
| Unknown | 3 (10.7%) | 0 (0.0%) | 3 (13.0%) |  |
| Previous therapy |  |  |  |  |
| PBC |  | 5 (100.0%) | 14 (60.9%) | 0.193 |
| MKIs | 12 (42.9%) | 4 (80.0%) | 8 (34.8%) | 0.121 |

ECOG PS, Eastern Cooperative Oncology Group Performance Status; PBC, Platinum-based chemotherapy; PD-1, Programmed death-1; MKIs, Multikinase inhibitors.

**Table 4. Baseline characteristics between patients with or without previous MKIs**

| Characteristics | Number (n=28, %) | Previous MKIs (n=12, %) | No previous MKIs (n=16, %) | P value |
| --- | --- | --- | --- | --- |
| Age (years) |  |  |  | 0.537 |
| <60 | 21 (75.0%) | 10 (83.3%) | 11 (68.8%) |  |
| ≥60 | 7 (25.0%) | 2 (16.7%) | 5 (31.3%) |  |
| Gender |  |  |  | 0.100 |
| Female | 15 (53.6%) | 9 (75.0%) | 6 (37.5%) |  |
| Male | 13 (46.4%) | 3 (25.0%) | 10 (62.5%) |  |
| Smoking history |  |  |  | 0.371 |
| Current or former | 8 (28.6%) | 2 (16.7%) | 6 (37.5%) |  |
| Never or unknown | 20 (71.4%) | 10 (83.3%) | 10 (62.5%) |  |
| Histology |  |  |  | 0.732 |
| Adenocarcinoma | 27 (96.4%) | 11 (91.7%) | 16 (100.0%) |  |
| Other | 1 (3.6%) | 1 (8.3%) | 0 (0.0%) |  |
| ECOG PS |  |  |  | 0.205 |
| 0-1 | 21 (75.0%) | 7 (58.3%) | 14 (87.5%) |  |
| 2 | 7 (25.0%) | 5 (41.7%) | 2 (12.5%) |  |
| Brain metastasis |  |  |  | 0.732 |
| Yes | 8 (28.6%) | 4 (33.3%) | 4 (25.0%) |  |
| No | 20 (71.4%) | 8 (66.7%) | 12 (75.0%) |  |
| RET fusion partner |  |  |  | 0.698 |
| KIF5B | 15 (53.6%) | 7 (58.3%) | 8 (50.0%) |  |
| CCDC6 | 5 (17.9%) | 2 (16.7%) | 3 (18.8%) |  |
| Other | 5 (17.9%) | 2 (16.7%) | 3 (18.8%) |  |
| Unknown | 3 (10.7%) | 1 (8.3%) | 2 (12.5%) |  |
| Previous therapy |  |  |  |  |
| PBC | 19 (67.9%) | 11 (91.7%) | 8 (50.0%) | 0.066 |
| Anti-PD-1 antibody | 6 (21.4%) | 4 (33.3%) | 1 (6.3%) | 0.241 |

ECOG PS, Eastern Cooperative Oncology Group Performance Status; PBC, Platinum-based chemotherapy; PD-1, Programmed death-1; MKIs, Multikinase inhibitors.
